# A case of hemorrhagic shock in a patient with neurofibromatosis type 1

**DOI:** 10.1002/ccr3.7013

**Published:** 2023-02-27

**Authors:** Yutaro Sakaguchi, Hiromu Okano, Ryosuke Furuya, Tsuyoshi Otsuka, Hiroshi Miyazaki

**Affiliations:** ^1^ Department of Emergency and Critical Care Medicine National Hospital Organization Yokohama Medical Center Yokohama Japan; ^2^ Department of Emergency Medicine, Graduate School of Medicine Yokohama City University Yokohama Japan; ^3^ International University of Health and Welfare Graduate School of Public Health Tokyo Japan

**Keywords:** hemorrhagic shock, neurofibromatosis type 1, shock

## Abstract

Complications of neurofibromatosis type 1 include fatal bleeding events due to vascular fragility. In this case of hemorrhagic shock due to a neurofibroma, the bleeding was controlled using an occlusion balloon and endovascular treatment which stabilized the patient. Systemic vascular investigation for bleeding sites is important to prevent fatal outcomes.

## Graphical abstract

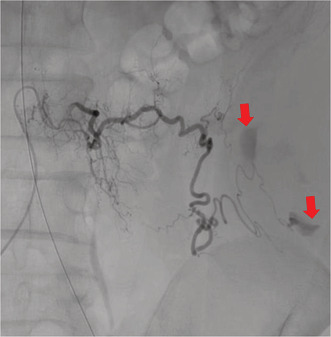

## 1 CASE PRESENTATION

**FIGURE 1:**
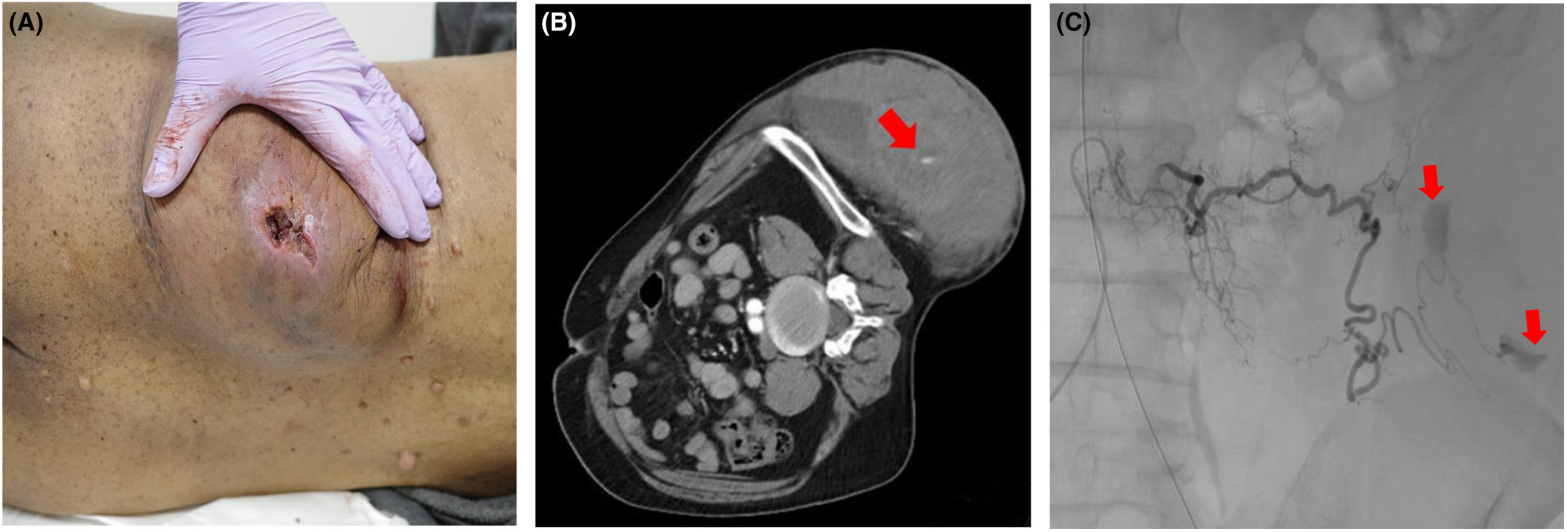
(A) Giant mass in the left lateral abdomen. (B) Contrast‐enhanced computed tomography revealed vascular leakage within the tumor (red arrow). (C) Emergency interventional radiology also showed vascular leakage (red arrows).

A 49‐year‐old man with neurofibromatosis type 1 (NF1) was brought to the emergency room because of swelling and pain in the left side of his abdomen. The patient had only recognized café‐au‐lait spots as symptoms of NF‐1. The mass on his lateral abdomen appeared upon awakening and was associated with severe pain, which led him to emergency care. He was in shock (pulse 112/min, blood pressure 112/75 mmHg, and coldness of limbs) when he arrived at the hospital, a large mass was observed on his lateral abdomen (Figure 1A). The hemoglobin level was 12.9 g/dL and there was no severe anemia. Circulation was unstable despite blood transfusion, and resuscitative endovascular balloon occlusion of the aorta (REBOA) was placed in ZONE I. Computed tomography findings showed an extravascular leakage within the tumor (Figure 1B), leading to the observed hemorrhagic shock. Emergency interventional radiology (IVR) was performed, and an extravascular leak was found from the left third lumbar artery, which was embolized with a gelatin sponge. The left first and fourth lumbar arteries were involved in the tumor and were embolized. (Figure 1C). A secondary finding was an aneurysm of the fourth lumbar artery. After the procedure, the patient was admitted to the ICU, and CT was repeated on days 2 and 7 without evidence of tumor growth. Strict blood pressure control was used to prevent rebleeding and rupture of aneurysms. Only one blood transfusion was given at the time of initial treatment, and there was no progression of anemia throughout the rest of the day. The patient was discharged on day 11. CT follow‐up was performed at 1, 3, and 6 months after discharge, and follow‐up was terminated when there was no tumor growth.

NF1 is an autosomal dominant inherited disease with an incidence of about 1:3000. In the present case, tumor hemorrhage was evident owing to an enlarged and painful lateral abdominal mass; additionally, an unruptured lumbar aneurysm was discovered. The incidence of vascular lesions in patients with NF1 has been reported to be 3.6%.^1^ In addition, vascular lesions in NF‐1 patients are reported to involve multiple vessels throughout the body.^2^ Since most patients with vascular disorders are asymptomatic, it is likely that there are many more patients with undiagnosed vascular disorders.

For infrequent complications of rare diseases, diagnosis and action are delayed. Therefore, when emergency room physicians encounter patients with NF1, they should be aware of hemorrhagic shock as a complication.

As for treatment, the patient was stabilized with endovascular treatment. Surgical hemostasis is reported to be difficult due to vascular fragility. In cases of hemodynamic instability, IVR can be safely performed with REBOA, as in this case.^3^

Vascular disorders are second only to malignancy in terms of cause of death in NF‐1 patients. Since multiple vessels can be involved, it is important to systemically investigate for vascular involvement to prevent fatal hemorrhage.

## AUTHOR CONTRIBUTIONS

YS wrote the first draft of the manuscript. HO, SN, HH, SY, TO, HM, and RF critically revised the manuscript. All authors read and approved the final version of the manuscript.

## CONFLICT OF INTEREST STATEMENT

The authors declare no potential conflicts of interest for this article.

## ETHICS STATEMENT

This article does not contain any studies involving human participants or animals.

## CONSENT

Written consent was obtained from the patient to disclose the case details and images pertaining to diagnosis.

## Data Availability

Data available on request from the authors.
